# Approach-aversion in calves following injections

**DOI:** 10.1038/s41598-018-27669-7

**Published:** 2018-06-21

**Authors:** Thomas Ede, Marina A. G. von Keyserlingk, Daniel M. Weary

**Affiliations:** Animal Welfare Program, Faculty of Land and Food Systems, University of British Columbia, Vancouver, B.C. Canada

## Abstract

We assessed aversion to injections using an avoidance-learning paradigm. Holstein calves (*n* = 24) were randomly assigned to one of four routes of administration for 0.5 ml of saline: intramuscular (IM), intranasal (IN), subcutaneous (SC) and a null control. Calves were first trained to approach a milk reward of 1 L. Once the latency to approach the reward was consistent, calves received their assigned treatment when approaching the bottle. For the first 3 treatment sessions calves received a 1 L milk reward. This reward was then reduced to 500 mL, and then to 250 mL, and finally to 0 mL, each for 3 sessions. Compared to control calves, calves receiving the intramuscular injections showed a longer latency to approach the milk reward, but only when the milk reward was 0.25 L (P = 0.05) and 0 L (P < 0.01). Calves receiving the intranasal injections showed longer latencies relative to the controls only for the 0 L reward (P = 0.01). Calves receiving the subcutaneous injections did not differ from controls for any of the milk rewards (P > 0.2). We conclude that IM injections are aversive and that SC and IN routes are a refinement to be considered when feasible.

## Introduction

Injections by needle are widely used in veterinary practice for the administration of medicine, vaccination and anaesthesia. The pain caused by injections has been studied in humans^1–5^ but little is known about how aversive these procedures are to animals. Research on acute noxious stimuli in animals often relies upon physiological indicators and behavior measures including withdrawal movements, writhing and defensive behaviors^6,7^. However, both approaches have questionable specificity^7^ and there are difficulties in drawing inferences regarding affect or motivation from such responses^8^.

Conditioning paradigms provide an alternative approach^9^. A multitude of experimental options exists^10^, some relying on the animals actively performing a behavior (such as pressing a lever, pecking a key, or jumping a barrier) to avoid an aversive treatment. However, animals may find it difficult to learn active responses in a stressful environment^11^. An alternative is to implement a passive avoidance paradigm in which animals are first trained to carry out a motivated behavior (such as accessing a food reward) that is then associated with a negative event. The animal can then choose whether they are willing to pay the price of enduring the negative event to gain access to the reward. The animal’s reaction to this conflict provides insight regarding motivational balance; i.e. the relative value allocated to the reward and the negative event. If the animal begins to avoid the treatment, it is reasonable to infer that the animal finds the treatment more aversive than it finds the food rewarding^12^. By providing control to the animal, this design also minimises other sources of distress^11^.

Conditioning paradigms have been used to assess a broad range of experiences, including those induced by drugs^13^, chronic arthritis^14^, electric shocks^15,16^, mechanical hyperalgesia^17^ and radiation^18^. More recently, with growing public concern over animal husbandry practices^19,20^, conditioning has been used to assess the aversiveness of procedures used for euthanasia^21,22^, cage-cleaning^23^ and handling^24^. To our knowledge no work to date has addressed aversion due to injections.

The purpose of this study was to assess the aversiveness of intramuscular (IM) injections, and to compare this with two possible refinements: intranasal (IN) and subcutaneous (SC). Calves were first trained to approach a milk reward. After training was completed, accessing the reward was paired with an injection and aversion was measured as an increase in the approach latency. The quantity of milk provided was gradually reduced to better assess the motivational balance between accessing the reward and avoiding the treatment. Intramuscular injections, and to a lesser extent subcutaneous and intranasal injections, were expected to increase approach latencies relative to control calves that received no injection.

## Methods

This study was conducted from January to June 2017 at the University of British Columbia Dairy and Education Center in Agassiz, British Columbia. The study was approved by the UBC Animal Care Committee (Application A16-0310) and performed in accordance with the guidelines outlined by the Canadian Council of Animal Care (2009)^25^.

### Animals, housing and treatments

24 Holstein calves were enrolled into the experiment at 22 ± 11 (mean ± S.D.) days of age. Calves were housed in groups of 10 in pens measuring 4.9 × 7.3 m bedded with 10 cm of sawdust. *Ad libitum* access to water and hay was provided through automatic feeders (RIC; Insentec B.V., Netherlands). A daily whole milk ration of 12 L was available through an automatic milk dispenser (CF 1000 CS Combi; DeLaval Inc., Sweden). Milk was not accessible in the 12 h before the test session began at 0900 h. The calves were returned to the group pen after the test session and received their full milk ration for the rest of the day.

Each calf was randomly assigned to one of four treatment groups: intramuscular (IM), intranasal (IN), subcutaneous (SC) or Control. Each treatment group received 0.5 mL of saline solution. IM and SC groups were injected in the rump with a 0.9 × 40 mm and 0.9 × 20 mm hypodermic needle respectively (Covidien 8881251766/8881251782). The IN group received the solution in the right nostril through a vaccination nasal tip (HTI Plastics, A003-A00-06).

### Apparatus

**Figure 1:**
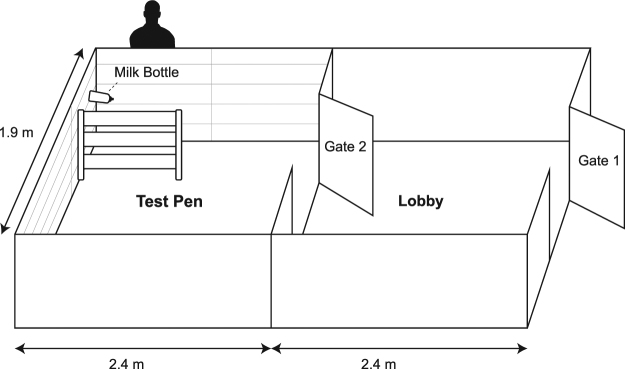
Experimental apparatus. Drawn by Shirley Ho.

Figure 1. The experimental apparatus was divided in two areas: the *Lobby* measured 1.9 × 2.4 m (4.6 m^2^), had unpainted plywood walls and concrete flooring covered with 10 cm of sawdust, and was accessible through *Gate 1*. On the side opposite to the entrance was *Gate 2*, allowing access to the *Test pen*. At the end of the Test pen (opposite to Gate 2) was a milk bottle with the teat positioned 80 cm above the floor. The Test pen was identical to the Lobby except that walls were mounted with colored panels, either white or red depending on the experimental phase. After bringing the calf into the lobby, the handler stood outside the apparatus next to the bottle and operated the gates remotely.

### Procedure and measurements

Each calf was tested individually; calves were moved gently from their home pen and brought to the lobby. Once in the lobby, the gate to the test pen (Gate 2) was opened and the calf was allowed to enter. Approach was characterized by the latency to start drinking the milk reward after Gate 2 was opened. If the calf did not approach the bottle within 5 min, the calf was brought back to her home pen and latency was recorded as 301 s.

### Habituation

During the habituation phase, 1 L of milk was placed in the bottle. Calves were allowed to approach and drink the reward without the administration of an injection. The panels mounted on the walls of the testing pen were white. Habituation ended once the calf spontaneously approached the bottle in less than 30 s and drank the full 1 L of milk, 3 d in a row.

### Treatment phase

Once the habituation was completed, the panels in the test pen were switched from white to red. This visual cue was introduced to facilitate the association. Previous work on Holstein calves has successfully used these same colors in a discrimination learning task^26^. During this phase, calves received their assigned treatments as soon as they started to drink from the bottle. The milk reward was decreased every 3 d from 1 L for the first 3 sessions, to 500 ml, then to 250 ml, and finally to 0 ml for the last 3 sessions.

### Statistical treatment

The three latencies obtained for each reward quantity were averaged to create a mean per calf per milk reward level. Data were log-transformed to normalize residuals. The transformed data were then modeled with a linear mixed-effects model^27,28^ that included as fixed effects the route of injection (3 df), the volume of milk reward (1 df) and the interaction (3 df). Calves were considered a random effect.

### Data availability

All data generated or analysed during this study are included available electronically.

## Results

During the last three days of habituation, calves assigned to the different treatments did not differ in latency to approach the bottle (F_3,20_ = 0.52, P = 0.7).

**Figure 2:**
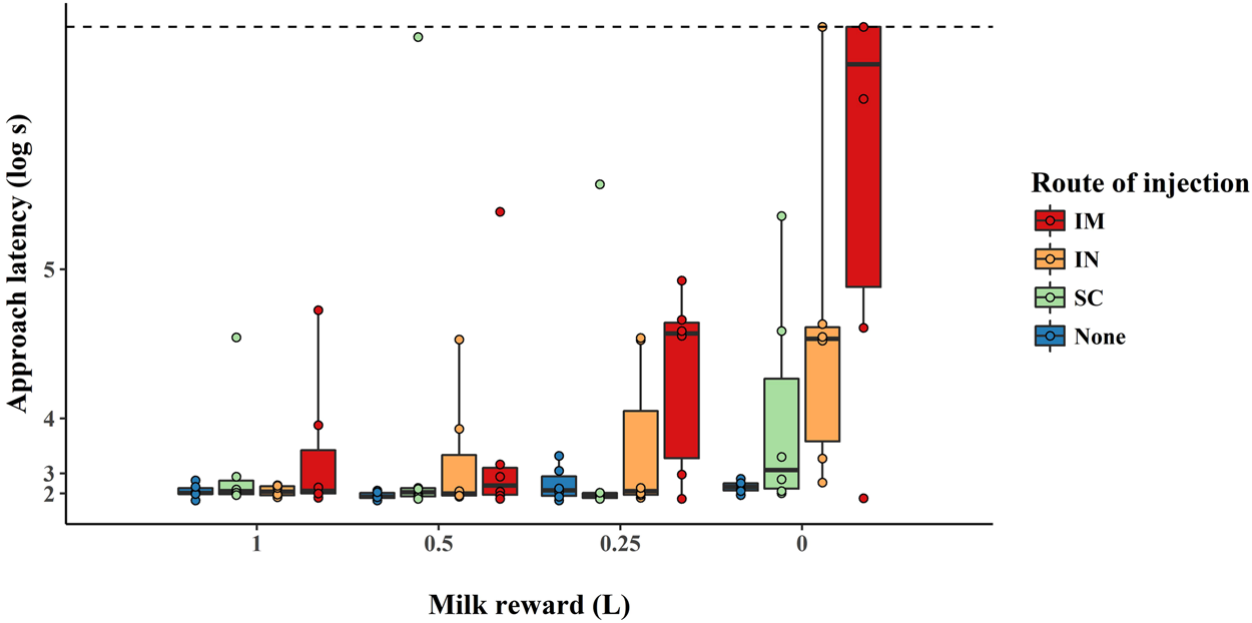
Latencies of calves to approach a milk reward, depending upon size of the reward and treatment (IM: Intramuscular, SC: Subcutaneous, IN: Intranasal). The latencies are log transformed and presented on an exponential transformed y-axis. The dotted line represents the latency limit.

The latency for calves to approach the milk bottle increased as the milk reward declined, but the magnitude of this decrease varied with treatment (Fig. 2), as indicated by the milk reward x treatment interaction (F_3,68_ = 3.3, P = 0.02). On the basis of this interaction, we analyzed the effect of treatment separately for each level of milk reward, using specified contrasts to compare each injection treatment with the control. None of the injection methods resulted in an increased approach latency relative to the control when the milk reward was 1 L (IM: t_1,20_ = 1.4, P = 0.2; IN: t_1,20_ = 0.1, P = 0.9; SC: t_1,20_ = 1.1, P = 0.3) or 0.5 L (IM: t_1,20_ = 1.5, P = 0.2; IN: t_1,20_ = 1.3, P = 0.2; SC: t_1,20_ = 1.2, P = 0.3). For the 0.25 L reward, only calves in the intramuscular treatment showed higher approach latencies compared to control calves (t_1,20_ = 2.1, P = 0.05). When no milk reward was provided, calves in both the intramuscular and intranasal treatments showed longer approach latencies relative to the control calves (IM: t_1,20_ = 3.4, P < 0.01; IN: t_1,20_ = 2.7, P = 0.01), with three animals reaching the 5 min threshold for the intramuscular group and one for intranasal. In contrast, the approach latencies for calves in the subcutaneous treatment did not differ from that of the controls (t_1,20_ = 1.4, P = 0.2).

## Discussion

When no treatment was administered, calves were highly and consistently motivated to access the milk reward. In contrast, groups receiving injections took longer to approach the reward, indicating a motivational shift from the approach of the milk to the avoidance of the procedure. The higher latencies recorded for the intramuscular treatment support our prediction that intramuscular injections are more aversive than the intranasal and subcutaneous routes.

The age of the animals was chosen to match that when dairy calves normally first experience injections (such as those associated with vaccination and disbudding). Adult animals also receive injections (for example, routine injections used in synchronized reproduction programs). Considering the development of thicker skin and adipose tissue as animals age, a stronger needle and greater force is required to pierce the tissue, perhaps affecting the relative aversion associated with different techniques. Similarly, different breeds and species may require different needles, forces, etc. Animals are also likely to vary in nose sensitivity affecting responses to the different treatments. Considering the frequent use of injections in veterinary practice, the literature is surprisingly sparse in regards to the aversion to the procedure and to potential refinements. Previous work has shown that 78% of dogs exhibit fear-related behavior on the examination table of a veterinary clinic^29^, and that 26% of dogs “yelp” when injected^30^, suggesting the need to consider less aversive methods. Aside from the obvious welfare implications of decreasing pain, better acceptance of injections may also facilitate handling, ensure a safer environment for the staff and likely improve the veterinary experience for the client^29^.

As an alternative to physical restraint, positive reinforcement techniques can be used to train animals to voluntary receive injections^31^. Although cooperation from the animal can be achieved for intramuscular injections, only half as many training sessions are needed for subcutaneous injections^32^.

In humans, verbal reports from patients show that intramuscular injections are more painful than the subcutaneous^33–35^ and intranasal alternatives^36,37^. Intramuscular injections cause tissue damage^38,39^, reach cells directly involved in inflammatory processes^40^, and may damage nerves^41,42^, whereas subcutaneous injections puncture only the epithelium. Pain is not limited to the puncture: injections of drugs such as antibiotics, anaesthetics and vaccines generally lead to more pain in humans when administered intramuscularly, which is thought to be linked to high innervation of muscle tissues^43^. Many species are known to have highly innervated muscle, so we expect our results with saline in calves to be consistent with various drugs and species. That said, we call for study of drugs on a case-by-case basis, as the aversiveness can be modulated by factors such as pH and volume of the solution injected^43^.

This study was limited to the treatment of the same volume through the different routes. However, the use of an alternative route might require a modified protocol to achieve similar efficacy. For example, intranasal treatments are not appropriate to all compounds as they must be able to cross the nasal epithelium^44^, but when applicable the intranasal route can provide higher efficacy^45^. A number of studies have reported similar efficacy when compounds were delivered via intramuscular and subcutaneous routes^46–48^.

The ability of avoidance paradigms to discriminate between treatments is sometimes limited by a ceiling effect causing “all or nothing” responses^12^. For example, 80% of rats have been observed to completely stop moving (i.e. “freeze”) when exposed to an environment associated with electric shock^49^. These all or nothing responses can be prevented through experiments eliciting conflict behaviors^50^, such that rather than only avoiding a negative event animals are also motivated to approach a positive one^11^. The difficulty lies in finding a conflicting situation that does not lean too much towards the positive (leading to the “all” response) or the negative (leading to the “nothing” response). Our results indicate that starting with a highly appetitive reward and then gradually reducing this, while keeping the treatment (i.e. the injection) constant, allows for a sensitive test of differences in aversion between treatments.

Multiple pairings may be required for animals to associate treatment and location. This means a difference in approach latency might have been observed between the treatment groups at earlier rewards if more training sessions had been allowed. The current study limited the number of sessions for ethical reasons (i.e. to limit the number of injections delivered to each animal), but studies using less invasive treatments may wish to consider more sessions at each reward level tested. By confounding the declining reward with time, our design made it impossible to distinguish between the effects of learning and the decline in reward size. It must emphasised that the existence of this confound was intentional; our aim was to compare treatments, not determine the precise quantity of milk calves were willing to give up to avoid each kind of injection. When the goal is to assess what quantity of reward is needed to overcome a negative event, it may be better to keep the reward constant^51^.

The results of the current study suggest that subcutaneous and intranasal routes are refinements over intramuscular injections. Pharmacokinetics and dynamics will vary with route of administration, but where feasible we recommend these alternatives to minimize aversion caused by injections.

## Electronic supplementary material

Dataset Approach-aversion in calves following injections
